# DNA Hyperstructure

**DOI:** 10.1021/acsomega.3c07379

**Published:** 2024-02-12

**Authors:** Gloria Elena León-Paz-de-Rodríguez, Ericka Rodríguez-León, Ramón Iñiguez-Palomares

**Affiliations:** † Independent researcher, Hermosillo C.P. 83250, Sonora, México; ‡ Physics Department, Universidad de Sonora, Hermosillo C.P. 83250, Sonora, México

## Abstract

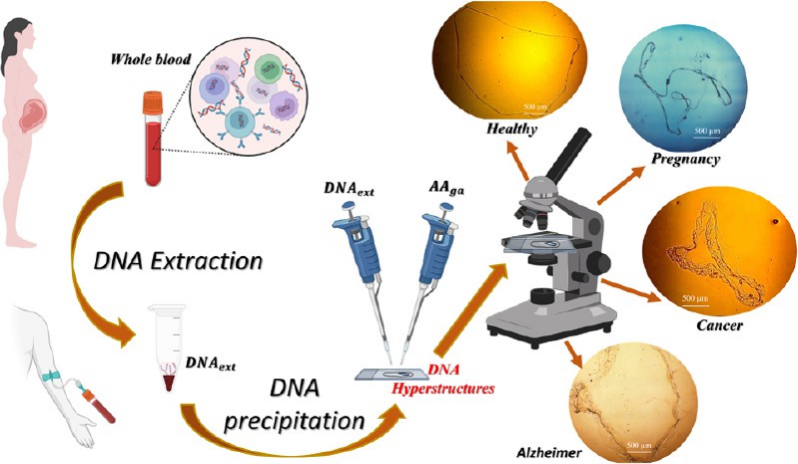

This study presents a new procedure to condense DNA molecules and precipitate them onto a glass slide. The resulting DNA molecules undergo autonomous self-assembly, creating closed superstructures on the micrometer scale, which are called DNA hyperstructures. These structures can be observed using low-magnification (4×) light microscopy. Precisely controlling the alcohol/glacial acetic acid ratio and DNA concentration during precipitation enabled the regulation of structure compaction on the slide. The alcohol/glacial acetic acid ratio is inversely proportional to the DNA concentration to achieve optimal compaction on the slide. Confocal microscopy fluorescence analysis of DNA extracts stained with DAPI shows that nucleic acids self-assemble to form structures during precipitation on the slide. This methodology is relevant since it facilitates the precipitation and visualization of DNA, regardless of its origin or molecular weight. To confirm its versatility, results with DNA extracted from human peripheral blood, the Lambda virus, and plasmid pBR322 are presented. The study examined the morphological features of DNA hyperstructures in both healthy individuals and those diagnosed with different medical conditions or illnesses, revealing distinct patterns specific to each case. This innovative technology has potential for disease detection in peripheral blood samples, ranging from cancer and Alzheimer’s disease to determining the gender of the gestational product at an early stage.

## Introduction

Diagnostics are essential in determining medical strategies. The COVID-19 pandemic highlighted the need for methodologies that allow for rapid and reliable diagnoses. The DNA molecule is composed of a deoxyribose alternating with phosphate groups, where each sugar is linked to one of four nitrogen-containing bases. In recent years, it has been shown that several diseases are related to changes experienced by the chemical environment of the DNA, which in turn translates into conformational modifications of the molecule.^1,2^ Various methods have been utilized for DNA research aimed at diagnostic purposes, among which is the examination of peripheral blood for the acquisition of circulating free DNA (cfDNA). Applications of diagnostic techniques using peripheral blood include numerous types of cancer, pregnancy, Alzheimer’s disease, and Down syndrome, as well as others.^3−5^ Cell-free DNA has been detected in blood through a quantitative polymerase chain reaction (qPCR). cfDNA originates from various tissues and possesses specific characteristics, including its size in base pairs. The condensed phase of DNA occurs naturally within cells and serves to shield molecules from external agents, preventing damage or mutations.^6^ DNA is a negatively charged molecule. Electrostatic shielding happens in environments with enough cations to cause condensed molecules.^7^ Condensed DNA is formed by reaching the isoelectric point, which happens when the molecule’s surface charge reaches zero and molecules associate to form larger structures. Pincus et al. conducted a study on this topic, finding that using spermine (a tetravalent cation) leads to structures of approximately 100 nm or even micrometers in size.^8^ In the laboratory, the condensation process is achieved through DNA oligomerization;^9−11^ the use of salts with mono-, di-, and tetravalent cations in varying concentrations has been studied;^12,13^ cationic surfactants^14^ and ethanol are also required to precipitate the DNA and create the conditions necessary for its self-assembly.^15^ In 1991, DNA was first observed under a light microscope (patent application filed in 1991 with the Mexican Institute of Industrial Property, granted in 1996)^16−18^ with the goal of finding an alternative method to replace electrophoresis. Methylation of the DNA molecule generates local structural changes in the double helix. Roll and propeller twist were the DNA shape features most sensitive to the methylation process.^19,20^ Modifications have also been detected in its mechanical properties,^21,22^ and changes in electronic properties and charge transport^23,24^ and ohmic resistance,^25^ changes (increase) in hydrophobicity due to methylation effects,^26,27^ and all changes have been observed to a greater or lesser extent when measuring these physical properties of the DNA molecule for different diseases. The diagnosis of diseases based on cytosine methylation has been reported for cardiovascular diseases, diabetes mellitus, cancer, and cerebral ischemia,^28^ among others. For example, a person with breast cancer will generate more or less surface electric charge than a healthy person, so the condensation conditions of the molecule will change, and therefore, the ideal experimental conditions must be established for this particular condition. The features in the DNA molecule to establish a diagnostic method have been studied by various analytical techniques: Cell-free DNA is studied in terms of chain fragmentation, i.e., shorter chains of a certain weight and longer chains are found. Fragmentation is associated with different diseases, as it can distinguish between cancer and healthy individuals.^29^ Another technique used is to measure the change in angle by attenuated total reflection, evaluating DNA methylation using fluorimetric, SERS, SPR, FRET, and colorimetric methods^30^ between healthy DNA samples and those of diseased individuals. Electrochemical method^31^ is another technique applied with the goal of diagnostics, studying differences between unmethylated and methylated DNA. Several studies confirm that changes occur in DNA at the structural level.^32^ In this study, we introduce a new technique for extracting DNA from peripheral blood and subsequently precipitating it onto slides. This method enables the creation of self-assembled DNA structures with millimeter dimensions that are reproducible under identical extraction and precipitation conditions. We present the results of our study on healthy individuals, pregnant women, and cancer patients, which reveal that the generated structures exhibit distinct morphological features.

## Experimental Section

### Materials

All chemicals and biological samples were purchased from Sigma and used as received without further purification. Trizma hydrochloride, molecular biology grade (BioUltra, ≥99%); sodium hydroxide, reagent grade (98%); ethylenediaminetetraacetic acid disodium salt dihydrate, molecular biology grade (BioUltra, ≥99.0%); phenol, molecular biology grade (≥99%); chloroform/isoamyl alcohol 24:1, molecular biology grade (BioUltra, ≥99.5%); absolute ethyl alcohol, molecular biology grade (≥99.45%); glacial acetic acid, ACS reagent grade (≥99.7%). Lambda Phage DNA and pBR322 Phage DNA were purchased as lyophilized powder.

### Methods

The BFC buffer was obtained by following a specific procedure for DNA extraction and precipitation. First, a 100 mL solution of 10 N NaOH in distilled water was prepared. Then, a solution containing 7.9 g of Trizma HCl, 7.5 g of EDTA, and 0.6 g of NaCl in 80 mL of distilled and sterile water was prepared to obtain the BFC buffer. After a pH range of 9.5–10 was achieved using the 10 N NaOH solution, it was added drop by drop, and the volume was adjusted with distilled and sterile water. Phenol was heated to 40 °C, and 15 mL of liquid phenol was obtained, which was then mixed with 10 mL of BFC through vortexing for 1 min at 2000 rpm. The mixture was refrigerated at 4 °C until use and separated into two immiscible phases. Phenol (P) was located at the top.

#### Protocol DNA Extraction

The study obtained informed consent from all participants prior to sample collection. Samples were collected from volunteer patients who had predetermined diagnoses, and the study followed the Helsinki Declaration. To collect the samples, 3–4 mL of peripheral blood was drawn into an EDTA-containing tube, incubated at room temperature for 24 h, and centrifuged at 3000 rpm for 5 min, and the resulting plasma was discarded. (1) Following the removal of plasma, the remaining material is identified as R and stored at a temperature of −20 °C until usage. (2) In a 1.5 mL tube, 4 μL of BFC and 40 μL of freshly thawed R are mixed and homogenized by vortexing for 5 s. Then, 10 μL of P is added and vortexed for another 5 s. Next, 2 μL of P and 2 μL of C (Chloroform + isoamyl alcohol 25:1) are introduced and homogenized by vortexing for 5 s. Finally, the mixture is centrifuged at 1000 rpm for 5 min. Afterward, 5 μL of phosphate buffer was added to the mixture, vortexed for 5 s, and then centrifuged at 1000 rpm for 30 min. The resulting DNA extract was obtained from the top layer.

### DNA Precipitation

**Figure 1:**
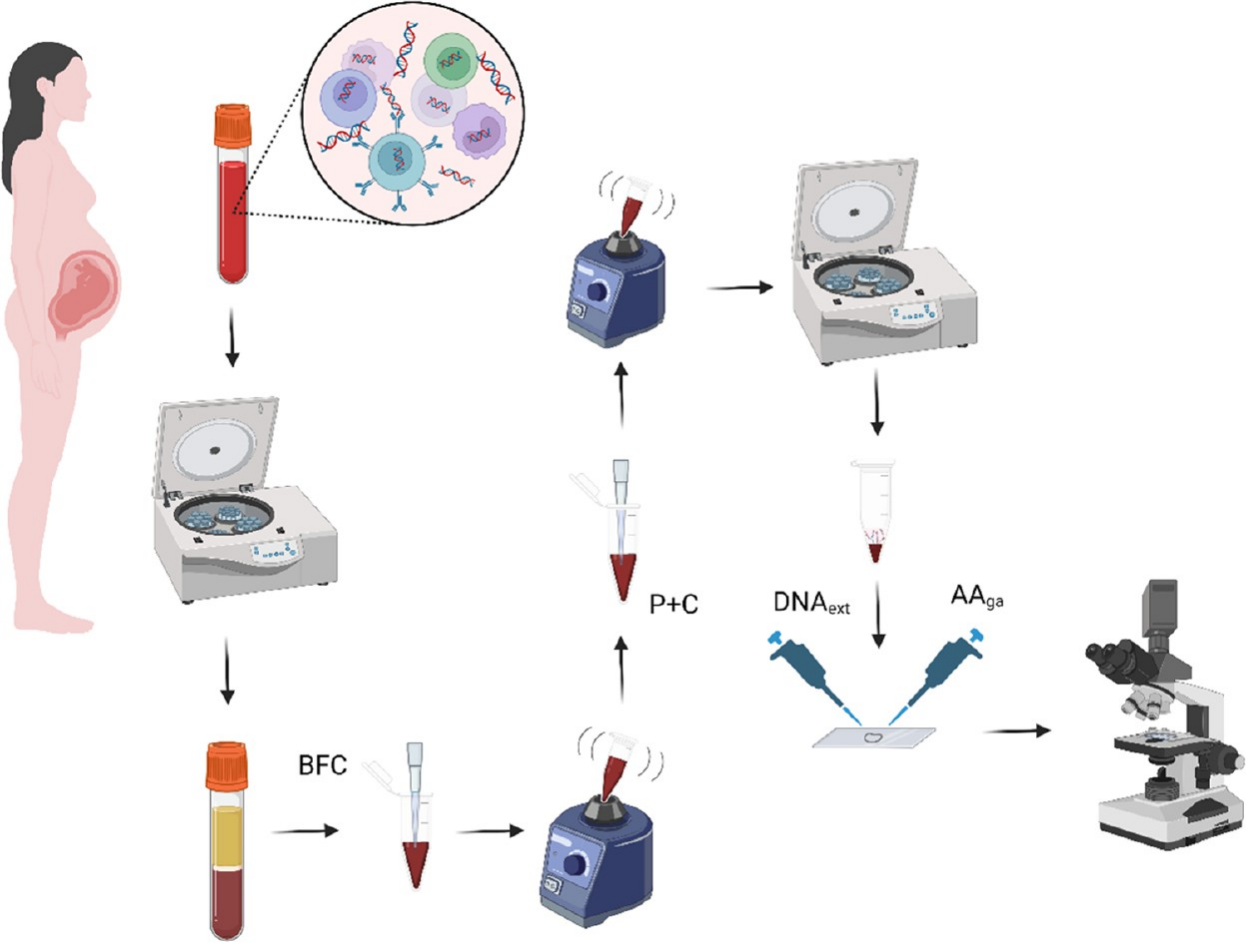
Summary of DNA extraction, condensed, and precipitation protocol. “Created with BioRender.com”.

(3) Take 1 μL of DNA extract (DNA_ext_) and precipitate it with 10 μL of AA_ga_ (absolute ethyl alcohol A + 5% glacial acetic acid A_ga_). These precipitation conditions are labeled as 1–10–5. Simultaneously deposit both agents on the slide. (4) Observed under a microscope (Figure 1).

### DAPI Staining for Confocal Microscopy

A 0.5 mg/mL DAPI solution was prepared in a BFC buffer. For 50 μL of DNA_ext_ sample (∼2 μg/μL), 1 μL of the DAPI solution was added and vortexed for 30 s. The sample was allowed to incubate in the dark at room temperature for 15 min, and then it was precipitated on slides. The volumes of DNA_ext_/DAPI and AA_ga_ ranged 0.5–2 μL and 7.5–25 μL, respectively. The percentage of A_ga_ in A ranged from 1 to 5%.

For confocal microscopy analysis, LSM800 equipment (Carl Zeiss, Jena Germany) mounted on an Axio Observer.Z1 inverted microscope (Carl Zeiss, Jena Germany) was used and with the objective of lower amplification mounted on the equipment (EC Plan-Neofluar 10*x*/0.3). A 405 nm laser with a maximum power of 5 mW was used as the excitation source, without exceeding 3.5% of the maximum value. For fluorescence detection, a high-sensitivity GaAsP detector was used and the bright-field image was obtained with laser light transmitted to a photomultiplier tube (PMT). For large work areas, the Tiles module of the Zen2 Blue Edition software (Carl Zeiss) allowed the acquisition of individual images and their merge into a mosaic of several photos, according to the required extension.

### DNA Extraction of Bloodstream: Condensation and Precipitation for Different Conditions

DNA hyperstructure of a healthy subject. DNA_ext_ was prepared as follows: 2 mL of peripheral blood was collected in a tube containing EDTA, incubated for 24 h at room temperature, then centrifuged at 3000 rpm for 5 min, the plasma was discarded, and the R residue was stored at −20 °C until use. For DNA_ext_, 40 μL of R was taken and transferred to a 1.5 mL tube containing 4 μL of BFC, vortexed, 10 μL of P was added, vortexed, 2 μL of P and then 2 μL of C were added, vortexed, and centrifuged at 1000 rpm for 5 min; finally 5 μL of P was added, vortexed, and centrifuged at 1000 rpm for 30 min. DNA_ext_ was then precipitated directly onto the slide by precipitating 1 μL of DNA_ext_ with 10 μL of AA_ga_ containing 5% A_ga_ (see Table S1).

DNA hyperstructure of patients with breast cancer and patients with uterine cancer were analyzed, DNA_ext_ was obtained with 0 μL of BFC in a 1.5 mL tube capacity, 44 μL of R was added, and it was homogenized in vortex, 2 μL of P was added, homogenized in vortex, 2 μL of P was added, and 2 μL of C was homogenized in vortex and centrifuged at 1000 rpm for 5 min. Then, 2 μL of P was added, homogenized by vortexing, and centrifuged at 1000 rpm for 30 min. Then, 0.4 μL of DNA_ext_ was taken and precipitated directly on the slide with 20 μL A containing 0.1% A_ga_ (see Table S2).

DNA of a subject with Alzheimer’s disease was extracted from 8 mL of peripheral blood, in an EDTA tube, and centrifuged at 3000 rpm, and 3.2 mL of plasma was taken, centrifuged at 4000 rpm, and the supernatant was discarded, the residue was resuspended with 20 μL of BFC and homogenized in a vortex, 80 μL of P and 160 μL of C were added and homogenized in a vortex and centrifuged at 10 000 rpm for 15 min. It was precipitated directly on the slide, and 1 μL of DNA_ext_ was taken and precipitated with 75 μL of AA_ga_ with 20% A_ga_.

DNA hyperstructure from an adolescent with Down Syndrome was analyzed in this study. DNA extraction utilized 25 μL of R, with the addition of 2.5 μL of BFC. The solution was homogenized in a vortex, followed by the addition of 25 μL of P and 25 μL of C. After further homogenization, the solution was centrifuged at 10 000 rpm for 15 min. 3 μL of DNA_ext_ and 25 μL of AA_ga_ with 30% A_ga_ were used for precipitation. The sample was observed under a microscope and photographed.

DNA hyperstructure of a pregnant woman results in the birth of a girl child. The DNA was extracted by combining 22 μL of P and 44 μL of R, followed by homogenization by using a vortex. Additional homogenization was performed using 1 μL of F and 1 μL of C, which were also homogenized in a vortex. The resulting extract was then centrifuged at 4000 rpm for 5 min, followed by a second centrifugation at 2000 rpm. After 60 min, 0.4 μL of DNA extract was precipitated with 8 μL of A and 0.1% A_ga_. The sample was observed under a microscope, and a photograph was taken (Table S3).

DNA hyperstructure of a pregnant woman from which a male child was born was extracted from peripheral blood using 22 μL of P and 44 μL of R, homogenized in a vortex, and then further homogenized using 1 μL of F and 1 μL of C, followed by centrifugation at 4000 rpm for 5 min and another round of centrifugation at 2000 rpm. For 60 min, 0.4 μL of DNA_ext_ was precipitated with 8 μL of A containing 0.1% A_ga_. Subsequently, it was observed under a microscope and photographed for further analysis (see Table S4).

## Results and Discussion

### DAPI Staining for Confocal Microscopy

**Figure 2:**
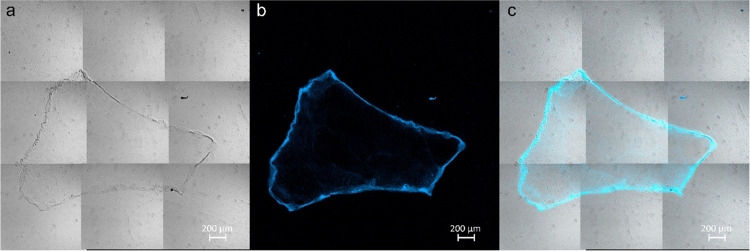
Images of DAPI-stained DNA_ext_ using confocal microscopy in (a) bright-field mode, (b) DAPI fluorescence, and (c) merged images.

A widely used strategy in fluorescent microscopy for the identification of nucleic acids is staining with DAPI (4′,6-diamidine-2′-phenylindole dihydrochloride). The DAPI stain associates with the minor groove of double-stranded DNA, with a preference for the adenine-thymine clusters.^33^ Once the DAPI–DNA coupling is formed, the emission of the dye is amplified with respect to its free coupling emission^34^ facilitating its detection by fluorescent techniques. In the present study, we used DAPI staining of DNA_ext_ obtained from circulating peripheral blood to demonstrate that the structures formed by precipitating the extracts on slides correspond to the self-organization of genetic material down to millimeter sizes. Figure 2 shows a sample of DNA_ext_ precipitated on slides with the ratio 1–10–5. Conditions of precipitation are defined: DNA_ext_ volume (μL), AA_ga_ volume (μL), percentage of A_ga_, and then (1–10–5) means 1 μL of DNA_ext_, 10 μL of AA_ga_ and AA_ga_ contains a 5% in volume of A_ga_. The sample corresponds to a 75-year-old woman diagnosed with breast cancer. In the bright-field image (Figure 2a), a closed structure stands out with a length of 1.96 mm of semimajor axis. Going around the perimeter, it is observed that the structure is made up of several strands or threads that in some regions join to form a compact and uniform strand that is darker than the rest with diameters between 4.5 and 10 μm. Interestingly, these compact strands can be fully stretched as at the top or forming “random coil”^35^-type structures as seen on the left side (see Figure S1). On the right side of the structure, we observe that the compact strand loses its homogeneity, and the constituent strands “open” or disperse to form ovoid structures that close at the other end to continue with the compact strand. This behavior is repeated every certain distance, breaking the continuity of the strand but completely closing the precipitated structure. The area bounded by the structure is 2.377 mm^2^. Figure 2b corresponds to the emission of the DAPI dye from the precipitated DNA sample. An intense emission is observed along the perimeter, indicating that the genetic material is found mainly in that region; therefore, the compact and scattered strands that are observed in the bright field are formed by the precipitated DNA and that it self-assembles forming these arrangements. To a lesser intensity, an emission is also observed inside the region delimited by the structure. Apparently, in the self-assembly process, not all of the molecules were integrated into the DNA hyperstructure formed. Figure 2c indicates the perfect splicing (merge) between the DAPI–DNA_ext_ emission and the structure formed in the bright field. To our knowledge, there are previous works with DAPI staining where DNA condensation by charge shielding is studied,^36,37^ and there are currently reports showing the formation of self-assembled DNA structures at millimeter scales.^38,39^ In this sense, in our work, we refer to structures with a characteristic pattern after precipitating the DNA on slides that can be systematically reproduced by applying an experimental protocol. Therefore, the term “DNA hyperstructure” is used to refer to the structures that are formed during the precipitation of DNA on a slide. Although it is not clear what is the precise mechanism that originates the precipitated structures, we maintain that some fundamental factors are the appropriate electrostatic shielding of the DNA strands by the presence of the salts in the concentrated buffer (BFC), the adequate value of the dielectric constant ε_r_ of the solvent during the precipitation on the slide, and the adequate relation between the concentration of DNA and the volume of AA_ga_ used in the precipitation.

**Figure 3:**
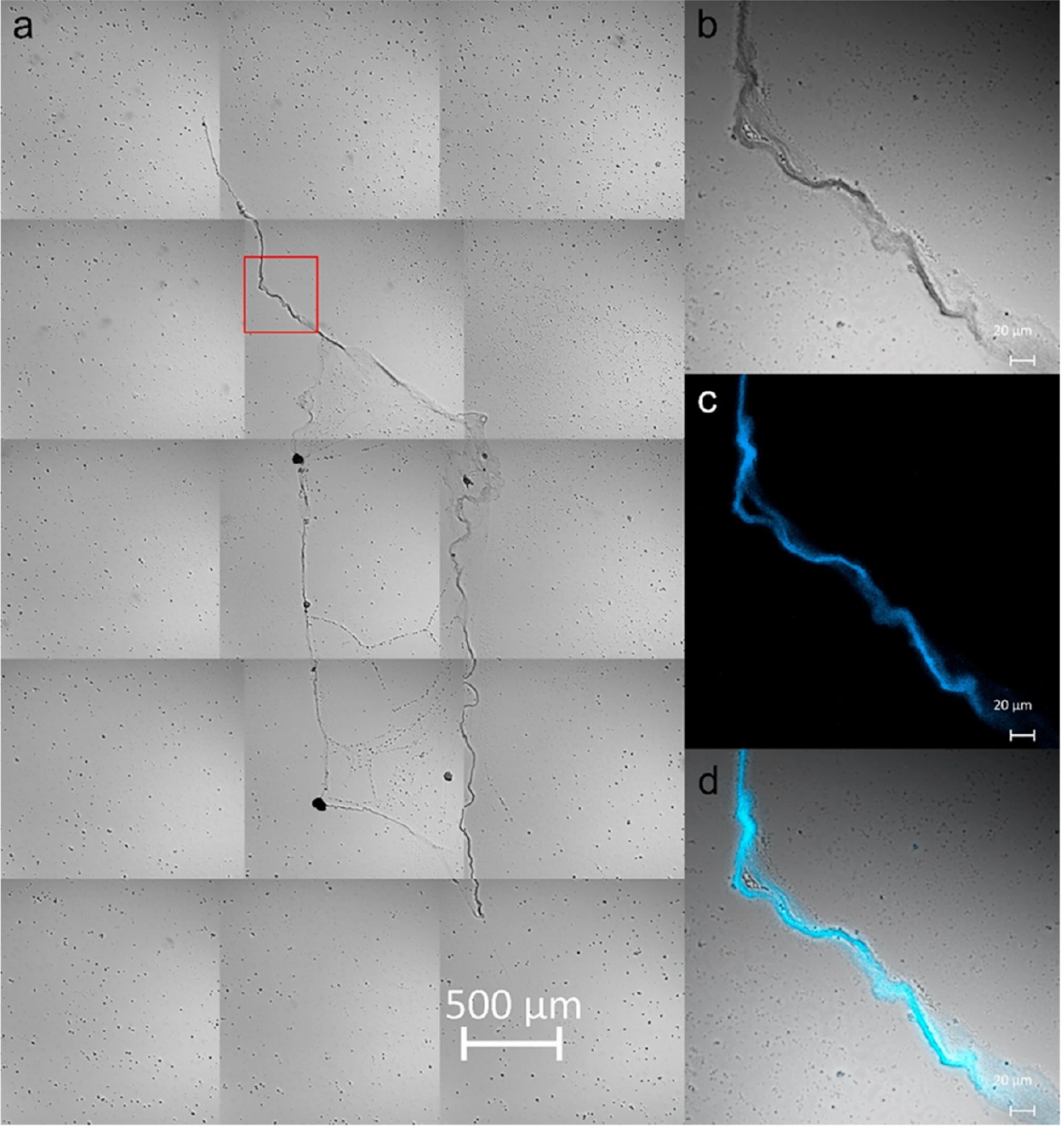
DAPI-stained DNA_ext_ images using confocal microscopy. (a) Bright-field mosaic image formed with 15 individual photographs obtained with a 10× objective. (b–d) High-resolution images of the red square delimited region in (a) that correspond to transmitted light or bright-field mode, DAPI fluorescence, and merged images, respectively. A 40× objective was used for zoomed images. The sample was precipitated on the slide with the 1–10–5 relation.

From the same DNA_ext_ analyzed above, another precipitation was performed for reproducibility purposes under the same conditions (1–10–5). Figure 3 shows the DNA hyperstructure obtained by the precipitation of the extract on slide. In the bright-field image (Figure 3a), a closed structure with a semimajor axis of 2.19 mm is observed. The elements in Figure 3 are similar to those in Figure 2. However, in the upper region of the image, there is an extension of the condensed DNA strand that measures 1.36 mm and extends beyond the enclosed area, which is the most significant and notable difference. The area bounded by this DNA hyperstructure is 1857 mm^2^. As will be seen later, the delimited area is the ideal parameter to compare the compaction of the DNA hyperstructure formed in the precipitation. For example, the value obtained for this second case is 80% of that of the previous area. Shown in red is the region where an image capture was performed at higher magnification (40×) as shown in the bright-field image (Figure 3b) and its corresponding DAPI fluorescence emission capture by confocal microscopy (Figure 3c). Both images are merged into Figure 3d. From these figures, the strands are formed by the nucleic acids of the DAPI-stained DNA precipitate, as evidenced by their intense and well-localized emission along the chain, as also shown in Figure S2.

**Figure 4:**
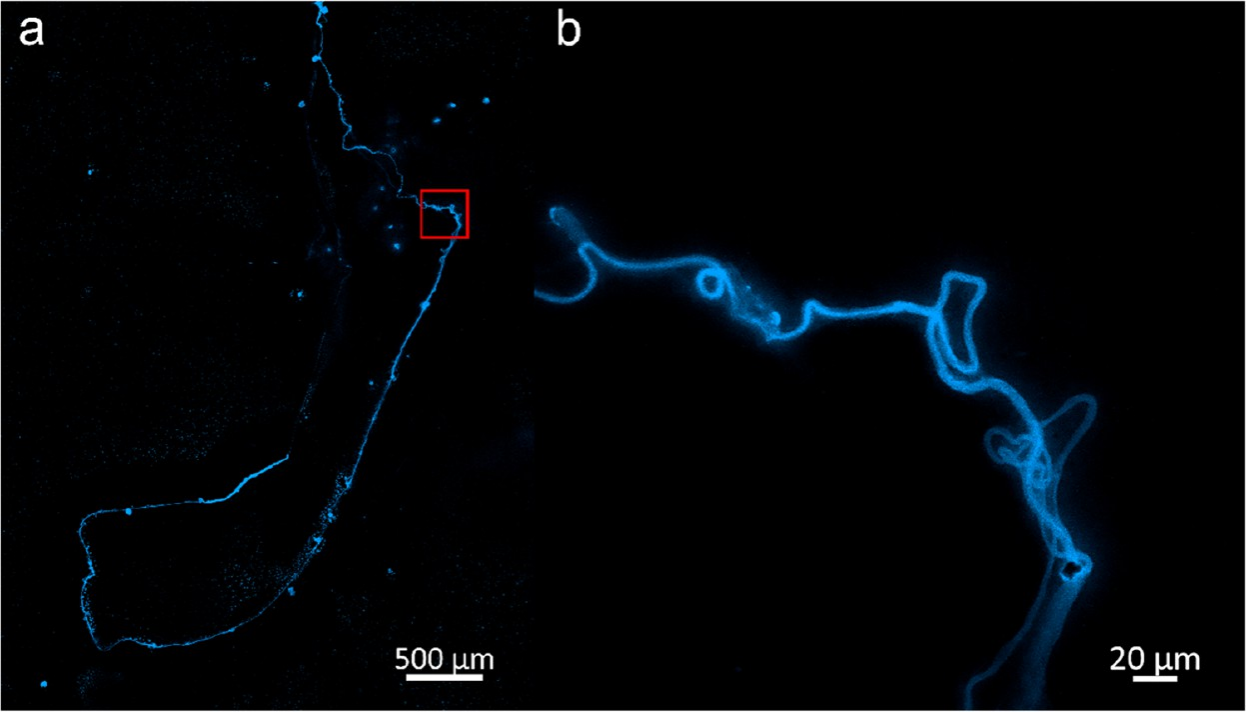
Confocal fluorescence images of DAPI-stained DNA_ext_. The sample was precipitated with the relation 1.5–15–1. (a) Mosaic image formed with 48 individual photographs obtained with a Plan-Apochromatic 40×/0.95 dry objective. (b) Individual image acquired at 40× of the selected region. For excitation, a 405 nm laser at 1.5% of the maximum power was used.

Figure 4 shows the DNA hyperstructure obtained with the precipitation condition of 1.5–15–1. In general, the structure is formed by an intense emission in the periphery that is mostly straight with little curvature (except in the upper part) and whose continuity is lost in the left part (Figures 4a and S3). A relevant aspect in the figure is indicated in the red box, which is amplified in Figure 4b. The image indicates that after the DNA molecules self-associate to form a compact strand or chain, this can fold or twist on itself without losing its integrity. In the upper part of Figure 4b, a well-defined strand of 3.5 μm in average diameter presents great flexibility, folding on itself twice. On the right side of the figure, the chain divides into other strands that emit with less intensity and with smaller diameters (2.5 μm).

### Condensed DNA Phase

**Figure 5:**
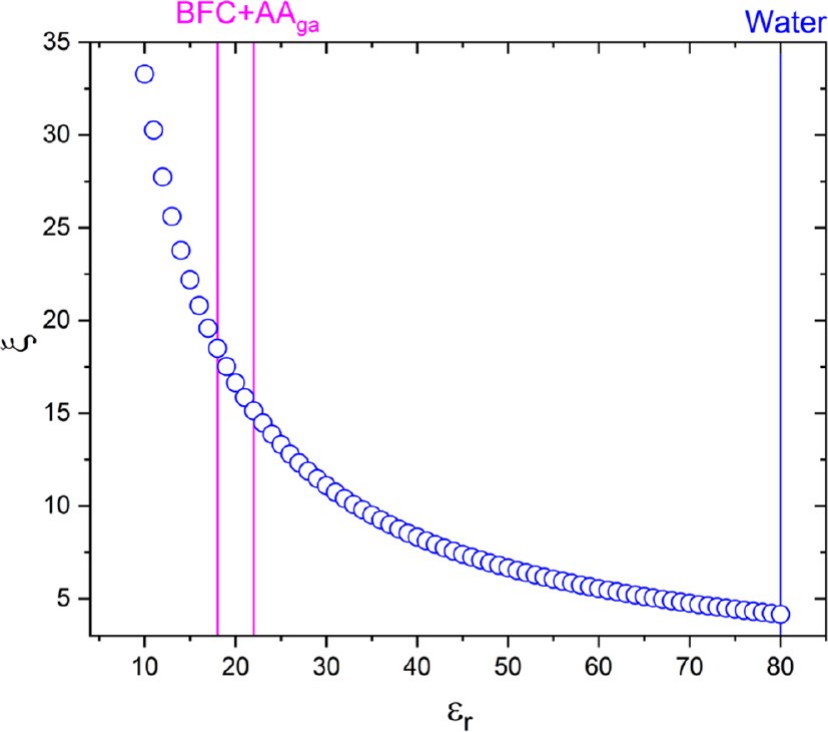
Charge coefficient of Manning as a function of ε_r_.

Buffer (BFC) used in this work is a buffer solution where the monovalent salt (NaCl) is 100 mM, and pH is around 10; these conditions are essential for the condensed DNA phase, reported by diverse authors.^40^ Similar results have been obtained by Shupeng He et al. using ethanol to precipitate DNA and salts for condensation; these salts can be monovalent as Na^+^, K^+^, divalent as Ca^2+^, Mg^2+^, and trivalent as Co^3+^, La^3+^, Al^3+^.^15^ Further, Carrivain et al. studied the condensation mechanism that generates a supercoiling molecule of DNA when monovalent ions have been used. Melnikov et al. demonstrated that the reduction of the dielectric permittivity of the solvent by the addition of primary alcohols to a dilute DNA solution promotes the compaction of individual DNA molecules. This effect is due to the increased electrostatic forces resulting from the decreased (dielectric permittivity of the solvent) ε_r_, which in turn increases the attraction between similarly charged monomers due to the increased ion–ion correlations.^41^ A study of the monovalent ion and interaction with DNA has determined the length value for which the interaction energies between two ions and thermal energy are equal;^42^ this depends on a dielectric constant relative to NaCl in water, Boltzmann constant (*K*_B_), and temperature (*T*) all in the international system of units. Equation 1 describes the Bjerrum length^43^
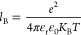
1where *e* is the proton charge and has a Bjerrum length of 7.06 Å at room temperature. Given that *b* represents the distance between the DNA molecule’s phosphates along the DNA axial axis^44−46^ and has a value of 1.7 Å, we calculate the ratio *l*_B_/*b* is 4.15, where this number is known as Manning fraction (ξ) and predicts an electrostatic screening when ξ > 1. When using water and AA_ga_, ε_r_ is obtained using the Onsager theory (2)
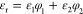
2where ε_1_ and ε_2_ are the dielectric constants for the solutions, and φ_1_ and φ_2_ are the fraction volumes. We used three solutions, A_ga_, A, and water + NaCl. Figure 5 shows the charge coefficient of Manning (ξ) as a function of the value of ε_r_ that changes as a function of Osanger eq 3, Θ is the number of contra-ions condensed for each phosphate group
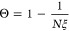
3where *N* is the valence of the salt solution; in our case, the values of ε_r_ vary between 15 and 20, and this increases the value of Θ to around 93–94%, which means that neutralization of charges is effective using the protocol proposed.

### Determination of AA_ga_ Content for the Precipitation of Condensed DNA_ext_

**Figure 6:**
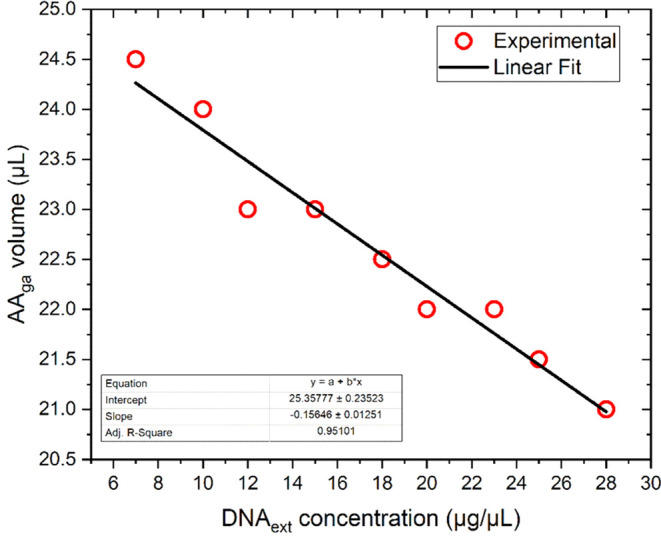
Calibration curve for AA_ga_ needed for optimal DNA hyperstructure formation as a function of DNA concentration in the precipitation process. Origin software.


The amount of AA_ga_ necessary for the precipitation of condensed DNA_ext_ was performed using a calibration curve. First, Lambda Virus lyophilized DNA was used to build a calibration curve by UV−vis spectroscopy and then determine the DNA concentration in the extracts obtained from peripheral blood. Condensed DNA_ext_ from a healthy female was used as a control, and their estimated concentration was 28 μg/μL. We obtained the linear fit equation from the calibration curve with the Lambda virus by UV−vis spectroscopy: *A*_260 nm_ = 0.01687 × *C*_DNA_. Subsequently, to measure the absorbance of the DNA_ext_ used as a control, 10 μL of DNA_ext_ was mixed with 2990 μL of BFC (total volume in the quartz cell was 3000 μL). The absorbance obtained under these conditions was *A*_260 nm_ = 1.586. Thus, multiplying by the dilution factor  gives DNA_ext_ = 28.2 μg/μL. Gong and Li mention that the conventional phenol-chloroform extraction method allows the recovery of an average of 4.5 μg of genomic DNA from 200 μL whole blood samples.^47^ Our method uses 4 mL of whole blood samples for DNA extraction. Theoretically, we would have 90 μg of DNA available in the extract obtained, so we consider the reported concentration range for the DNA_ext_ to be affordable. Subsequently, a new calibration curve relating the volume of AA_ga_ needed to precipitate DNA_ext_ on the slide adequately was constructed. For this, dilutions of DNA_ext_ were made, and for each concentration, the AA_ga_ volume that formed the optimal DNA hyperstructure in the precipitate was sought. In the precipitation tests, the volume of DNA_ext_ was kept constant (1 μL). The optimal DNA hyperstructure was considered to cover most of the visual field allowed by a 4× objective in a conventional optical microscope, as illustrated in Figure S4. The graph shown in Figure 6 was constructed with the optimal volumes for each concentration. The behavior of the straight line that fits the experimental data shows a negative slope. This indicates that the higher the DNA concentration, the smaller the volume of AA_ga_ required for adequate precipitation on slides. Diluted samples will require larger volumes of AA_ga_ to be properly observed when precipitating. The explicit dependence of the AA_ga_ volume required according to the concentration of DNA_ext_ is summarized in the following linear relationship, eq 4

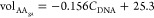
4

Furthermore, He et al.^15^ found that in DNA solutions with concentrations near 1 μg/μL and monovalent salts (100 mM), ethanol at a concentration of 60 vol % led to almost complete precipitation of DNA. In our study, we utilized AA_ga_ proportions for DNA precipitation ranging from 80 to 95% by volume, guaranteeing optimal conditions for efficient precipitation.

### Controlling the DNA Hyperstruture Compaction

**Figure 7:**
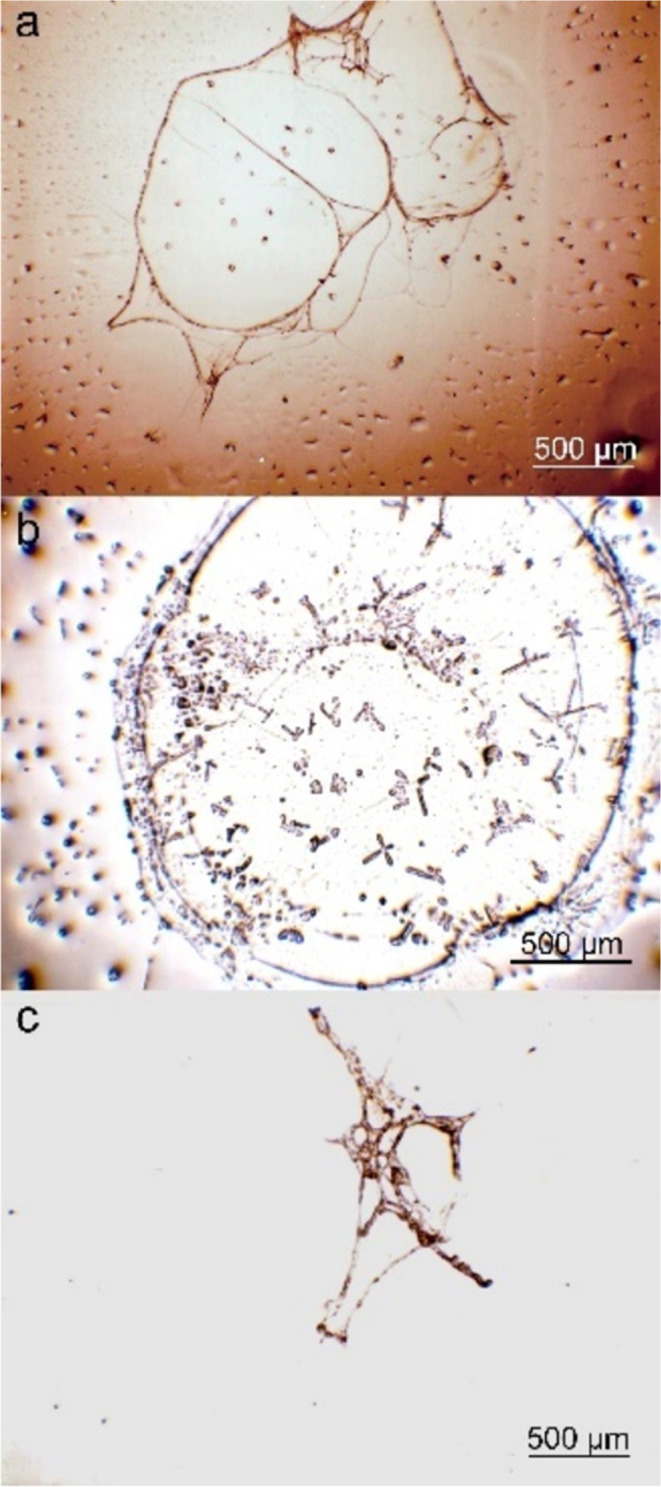
Lambda virus DNA was precipitated in the following proportions: (a) 1–1000–5, (b) 1–200–5, and (c) 1–100–5. Images acquired by optical microscopy and 4× objective.

We have found that a fixed concentration and volume of DNA_ext_ and the amount of AA_ga_ used during precipitation are the variables that regulate the compaction of the DNA hyperstructure obtained on the slide. To study the effect of AA_ga_ on compaction, Lambda virus lyophilized was acquired and used as a control, 50 μg of Lambda virus DNA was dissolved in 100 μL of BFC. Then, 1 μL of Lambda virus DNA was precipitated with 1000, 200, and 100 μL of AA_ga_ (95–5% v/v). Figure 7 shows (a) extended DNA hyperstructure, (b) circular DNA, and (c) compacted DNA of Lambda virus. The higher AA_ga_ content causes a more extended precipitate and compaction is achieved with the lower AA_ga_ content. Relevantly, these results indicate that the methodology proposed in this work also allows obtaining DNA hyperstructure from sources other than human DNA. Figure S5 shows the DNA hyperstructure obtained from plasmid pBR322. This cloning vector was purchased freeze-dried from a commercial company (Sigma) and dissolved in BFC under the same conditions as those for the Lambda virus. Surprisingly, the circular morphology of pBR322 DNA hyperstructure coincides with that reported by TEM.^48^

**Figure 8:**
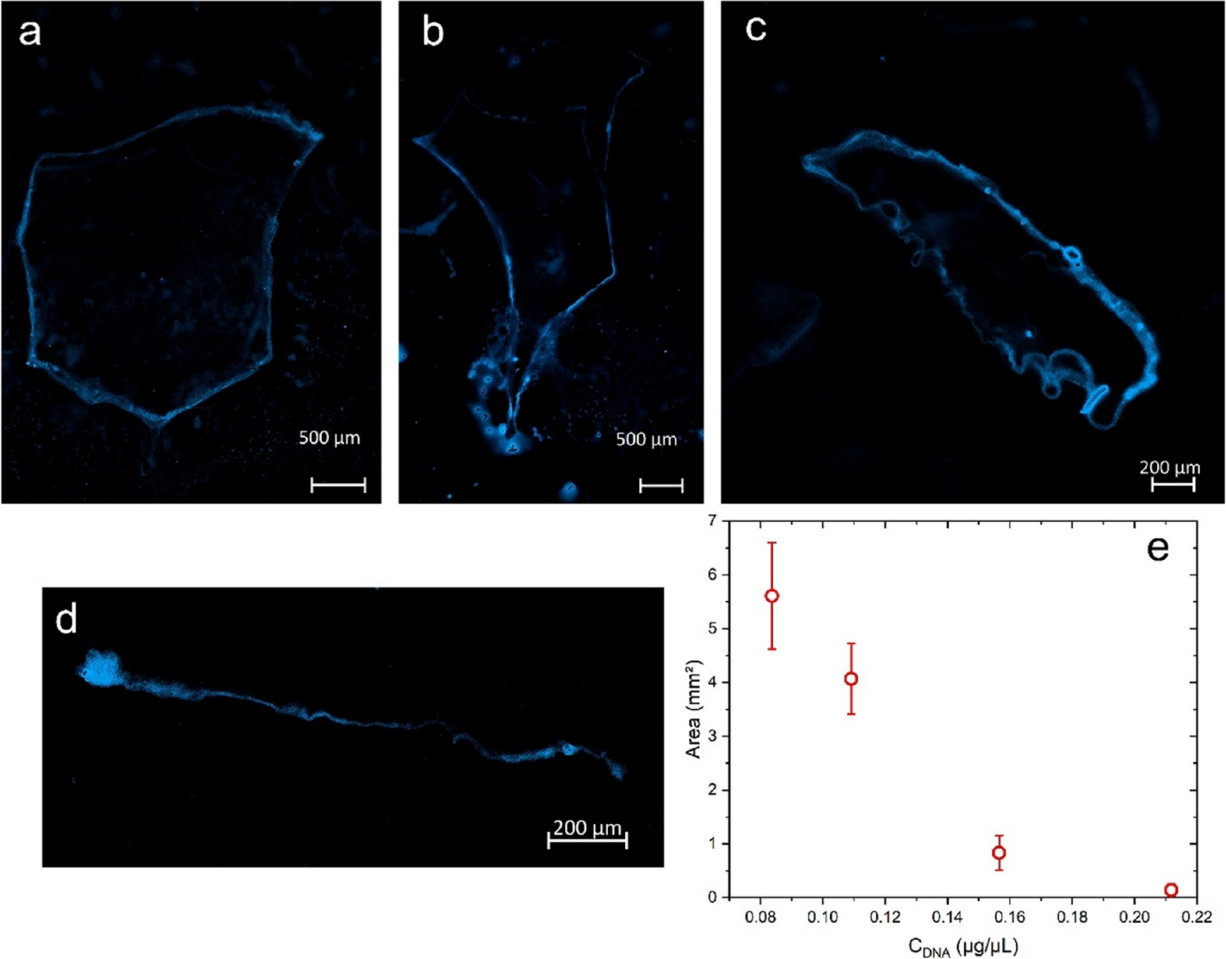
Photographs of DAPI-stained DNA hyperstructures on slides from the same sample and different precipitation conditions. The volume of DNA_ext_ was constant (1 μL), and the volumes of AA_ga_ were 20, 15, 10, and 7.5 μL in (a–d), respectively. (e) Behavior of the area delimited by DNA hyperstructure when varying the AA_ga_ content in the DNA_ext_ precipitation.

The study of DNA compaction was extended to peripheral blood samples. The images in Figure 8 correspond to the same DNA_ext_ sample at 1.8 μg/μL precipitated on slides with different proportions. In tests, the DNA_ext_ volume (1 μL) and A_ga_ content in the alcohol (1% v/v) were kept constant. The AA_ga_ volumes were 20, 15, 10, and 7.5 μL generating the structures shown in (a–d), respectively. Figure S6 shows the bright-field images merged with the fluorescent image associated with Figure 8. The perimeter-bounded area of the DNA hyperstructures was measured by using ImageJ software. We define the DNA concentration in the precipitation as an appropriate parameter to compare the effect of AA_ga_ on the DNA hyperstructure compaction. The volume of the solution is given by the DNA_ext_ volume + AA_ga_ volume, in each case. The DNA_ext_ content did not vary. Thus, Figure 8e shows a graph of the area about the DNA concentration. It is observed that there is a monotonous decreasing behavior of the area as the concentration increases. Therefore, the decrease in the AA_ga_ content during the precipitation of the sample on the slide generates more compact DNA hyperstructures.

### DNA Extraction of Bloodstream: Condensation and Precipitation for Different Conditions

**Figure 9:**
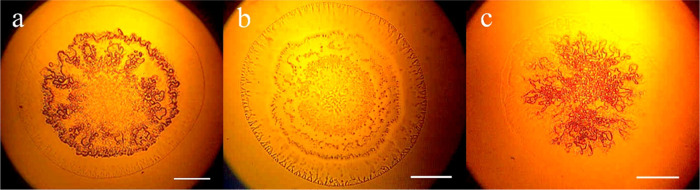
Dried DNA_ext_ (1 μL) without AA_ga_ at room temperature. Images (a) and (c) correspond to healthy subjects and image (b) corresponds to a pregnant woman. Scale corresponds to 500 μm.

The formation of DNA hyperstructures is closely tied to the solvent-drying process on the slide. Sufficient AA_ga_ is crucial for enabling DNA molecules self-assembly during slide precipitation. Without AA_ga_, DNA hyperstructure formation is unattainable.Figure 9 illustrates 1 μL of DNA_ext_ deposited on AA_ga_ free slides. In this scenario, the formation of DNA hyperstructures does not occur, resulting in the anticipated ring-like structure that is characteristic of a drying process involving a particle or macromolecule-filled solution drop, commonly known as the “coffee ring pattern”. The solvent in this case is solely composed of BFC, within which DNA_ext_ is dispersed.

When adding AA_ga_ during DNA_ext_ precipitation on the slide, we should expect a ring-shaped formation as the solvent evaporates. Surprisingly, the self-assembled structures present geometries far from the circular shape, and long straight extensions and other curved regions are observed on their perimeter (Figure S7). This suggests that within the DNA hyperstructure, there exist domains with varying mechanical properties. Specifically, the straight regions exhibit higher rigidity, while the remaining regions display greater flexibility.

**Figure 10:**
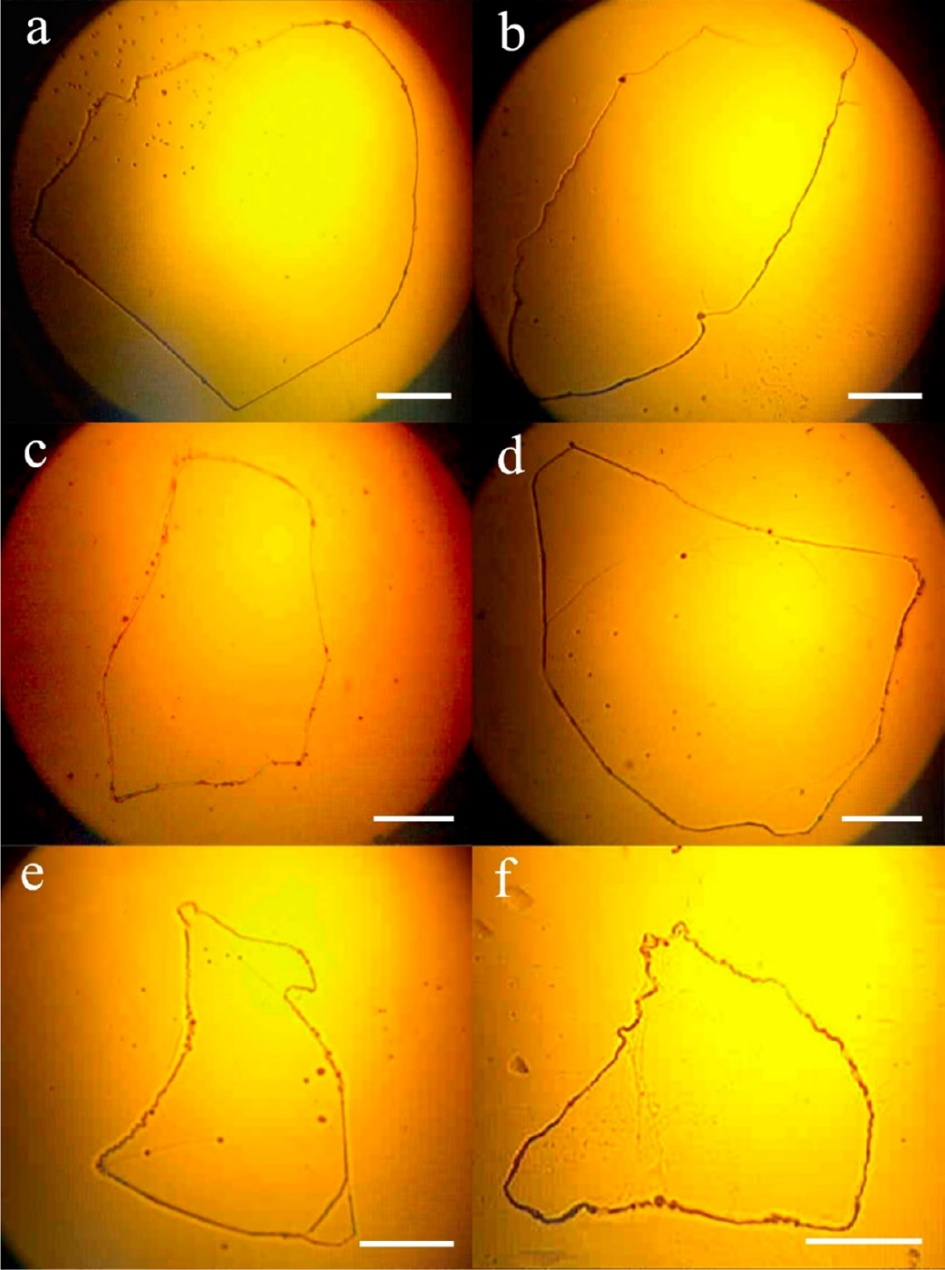
Hyperstructure of (a–f) DNA in healthy subjects under precipitation condition 1–10–5. The image was obtained by light microscopy using a 4× objective. DNA_ext_ and the precipitation protocol were the same for all samples. The scale bar in all images is 500 μm.

Figure 10 depicts the DNA hyperstructure of healthy individuals of different genders and ages. The extraction and precipitation conditions are the same for all cases. The precipitated structures exhibit a uniform morphology characterized by a single closed chain with a well-defined and uninterrupted perimeter featuring extended straight segments. Notably, the patterns generated exhibit strikingly similar morphologies, despite originating from different subjects, who share the common characteristic of not having chronic degenerative diseases.

**Figure 11:**
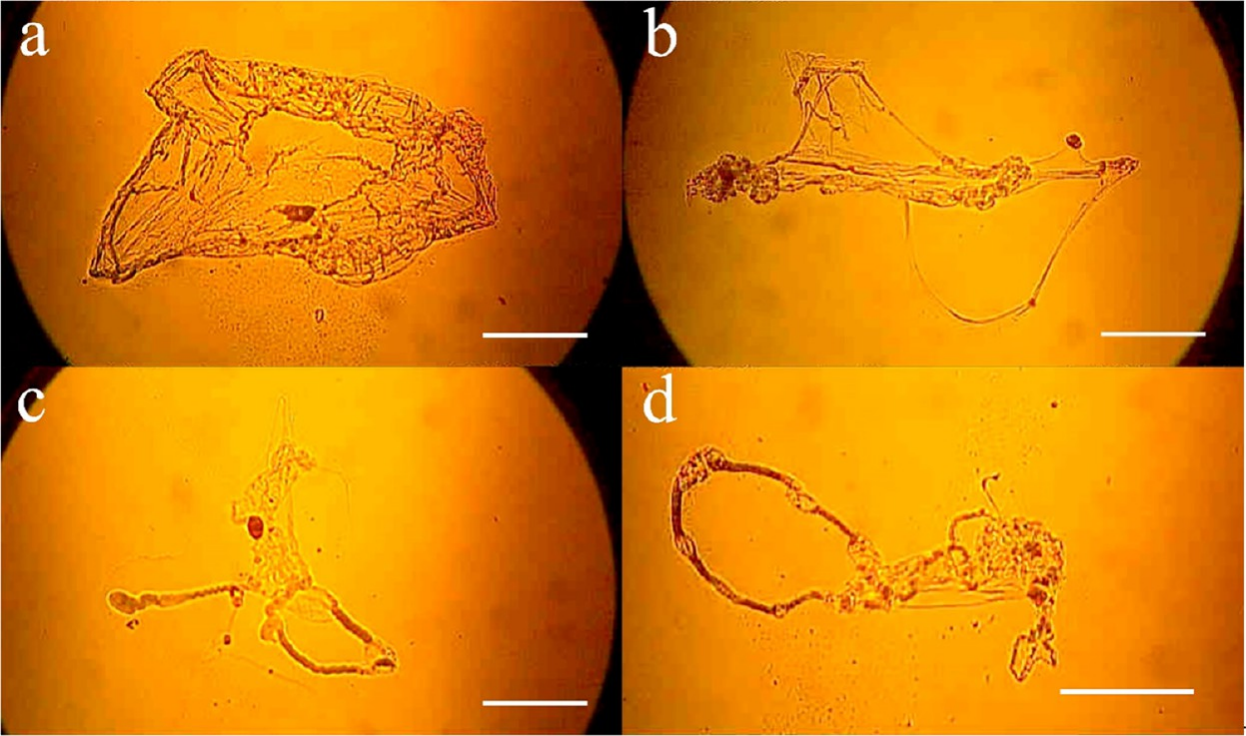
(a, b) DNA hyperstructure in breast cancer subjects with treatment and (c, d) uterine cancer subjects without treatment. All samples underwent the same precipitation conditions of 0.4, 20, and 0.1. Images were acquired by using optical microscopy and a 10× objective. The scale corresponds to 200 μm for all images.

Figure 11 shows the DNA hyperstructure of patient with (a, b) breast and (c, d) uterine cancer. The same protocol was used for DNA extraction and precipitation; in Figure 11(a), patient with preview chemotherapy and radiation therapy treatment, and in Figure 11(b), patient surgery and radiation therapy show a DNA hyperstructure with changes with respect to healthy subjects.

When applying the extraction protocol to both healthy individuals and patients with various conditions, we acquired images of DNA hyperstructures that exhibit noticeable modifications in morphology compared with those from healthy patients. Figure 11c,d illustrates images from uterine cancer patients lacking treatment, which differ from breast cancer images but bear similar features to one another. Cancer creates unique physical conditions that necessitate modifications to the protocol to identify the precise conditions necessary for the condensed and self-assembled DNA to form a DNA hyperstructure. It has been widely cited that cancer cell DNA has been detected in peripheral blood,^5,49−52^ quantifying the increase in cfDNA concentration has been utilized as a diagnostic method for breast cancer.^53^ Tumoral cells release two types of DNA, the first with information about the tumor, circulating tumor DNA (ctDNA), and healthy DNA.^54,55^ Atomic force microscopy images of methylated DNA from a cancer patient and healthy patients have been reported in several works.^56,57^ Similarly, a study of the structural changes of DNA due to the effect of methylation, which generates a process called fragmentation of cell-free cfDNA in circulation, shows important differences between healthy and sick individuals. Several methods have been reported for the isolation of cfDNA to analyze the sizes of each molecule. Based on these data, the sizes of molecules (in terms of base pairs) found in a characteristic size in healthy individuals are compared. These molecule sizes are found to be distinct and unique to individuals with different types of cancer and are obtained through PCR sequencing. Furthermore, a statistical study is conducted to determine the size of the DNA fragments.^29,58^

In our case, the extraction and precipitation of the DNA molecule includes all of the molecules present in the peripheral blood, so we observe a mixture of different types of DNA in a large structure. In our methodology for DNA hyperstructure, unlike in healthy patients where we observe an organized structure, here we see structures that lose the ability to organize the self-assembled structure, giving rise to a disorganized structure; we speculate that this may be due to methylated sites that prevent self-assembly.

**Figure 12:**
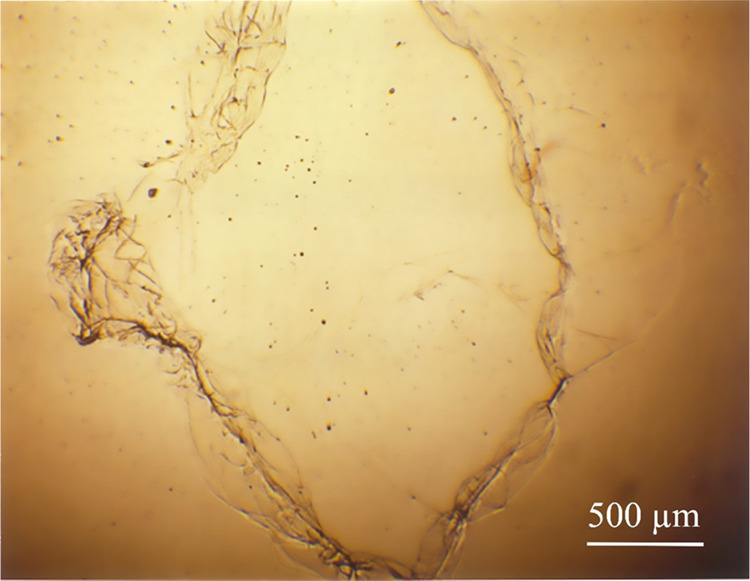
Alzheimer patient DNA hyperstructure. Precipitation condition 1–75–20. Image acquired by optical microscopy and 4× objective.

In Figure 12, we can see the DNA of a patient with Alzheimer’s disease. The image illustrates an incomplete self-assembly process of DNA chains in which several strands combine in a disorganized manner to form a diffuse perimeter. The ability to form self-organized DNA structures during the slide precipitation process seems to be impacted in patients with chronic illnesses. Studies report that brain damage observed in autopsies of people with Alzheimer’s and Down syndrome are similar, which suggests that they are the same microorganisms,^59^ or the same causative agent of both diseases.^60−66^ Other researchers observed alterations in the structure of DNA in Alzheimer’s patients by examining modifications in the DNA of mouth cells, which were analyzed using super-resolution microscopy (Figure 12).^67^

**Figure 13:**
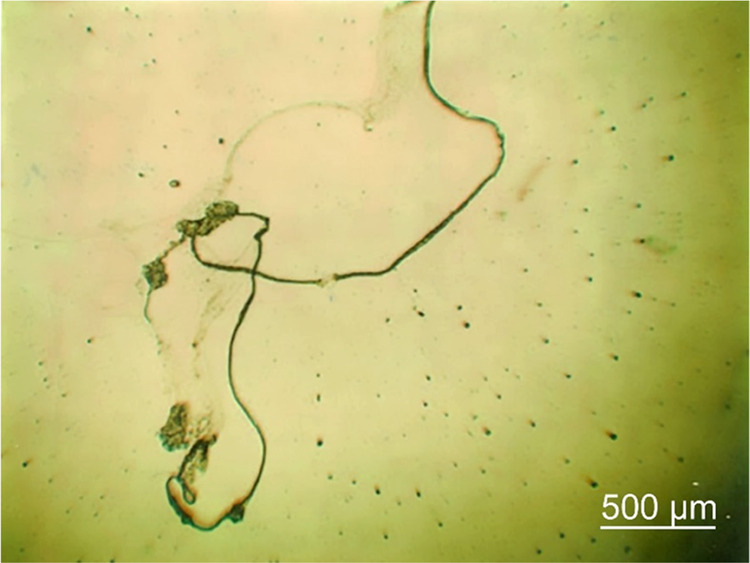
DNA hyperstructure from a teenager with Down syndrome. Precipitation condition 3–25–30. Image acquired by optical microscopy and 4× objective.

Figure 13 shows the DNA hyperstructure from an adolescent with Down syndrome.

**Figure 14:**
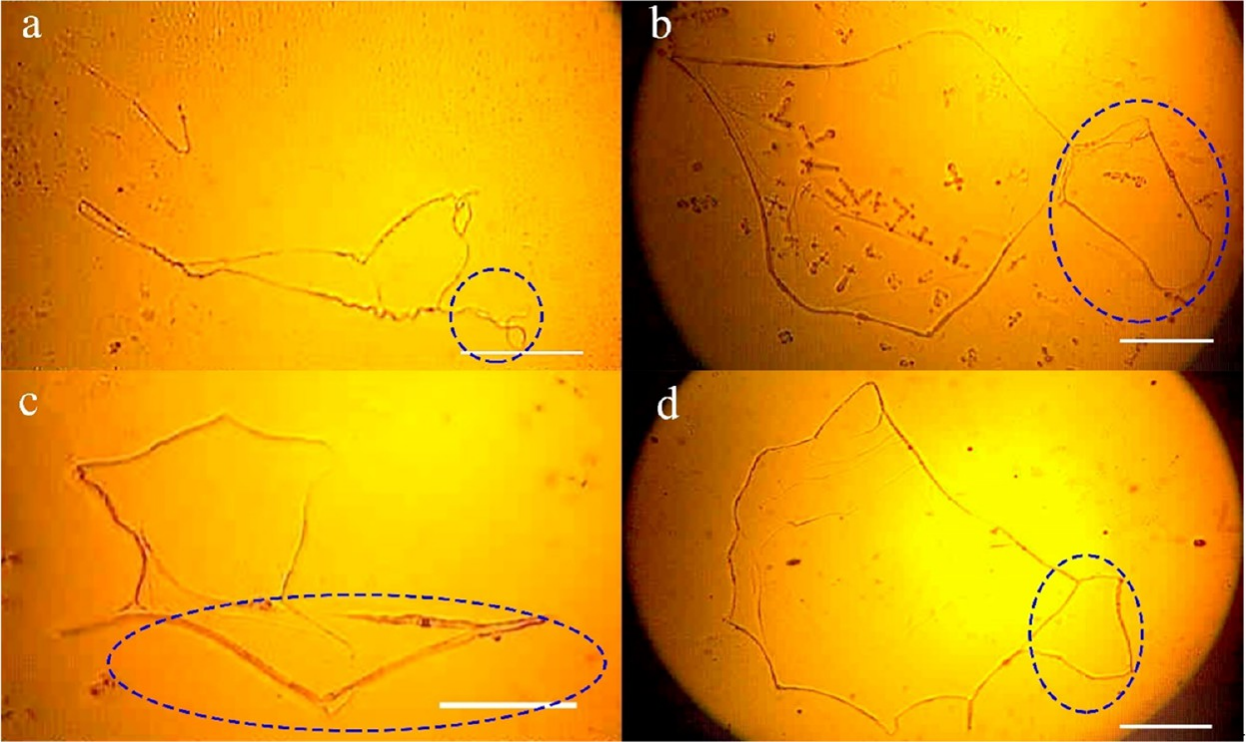
DNA hyperstructure of female fetuses at four different stages of gestation, specifically at 5, 13, 17, and 19 weeks. The precipitation conditions were (a, b) 0.4–10–0.1, (c) 0.4–5–0.1, and (d) 0.4–8–0.1. An optical microscopy image acquired using a 4× objective was utilized for the analysis. For all images, the scale corresponds to 500 μm.

In Figure 14, the DNA hyperstructure was analyzed in four different women who were pregnant at 5, 16, 17, and 19 weeks. In all cases, a small adjacent strand is observed on the outside of the main strand. Analysis of cfDNA is used for standard prenatal screening diverse reports established: maternal blood is used for the analysis of cfDNA, between 10 and 20% correspond with cfDNA of the fetus, is possible the diagnostic of trisomy 21 and other features of the fetus as gender.^68−70^

**Figure 15:**
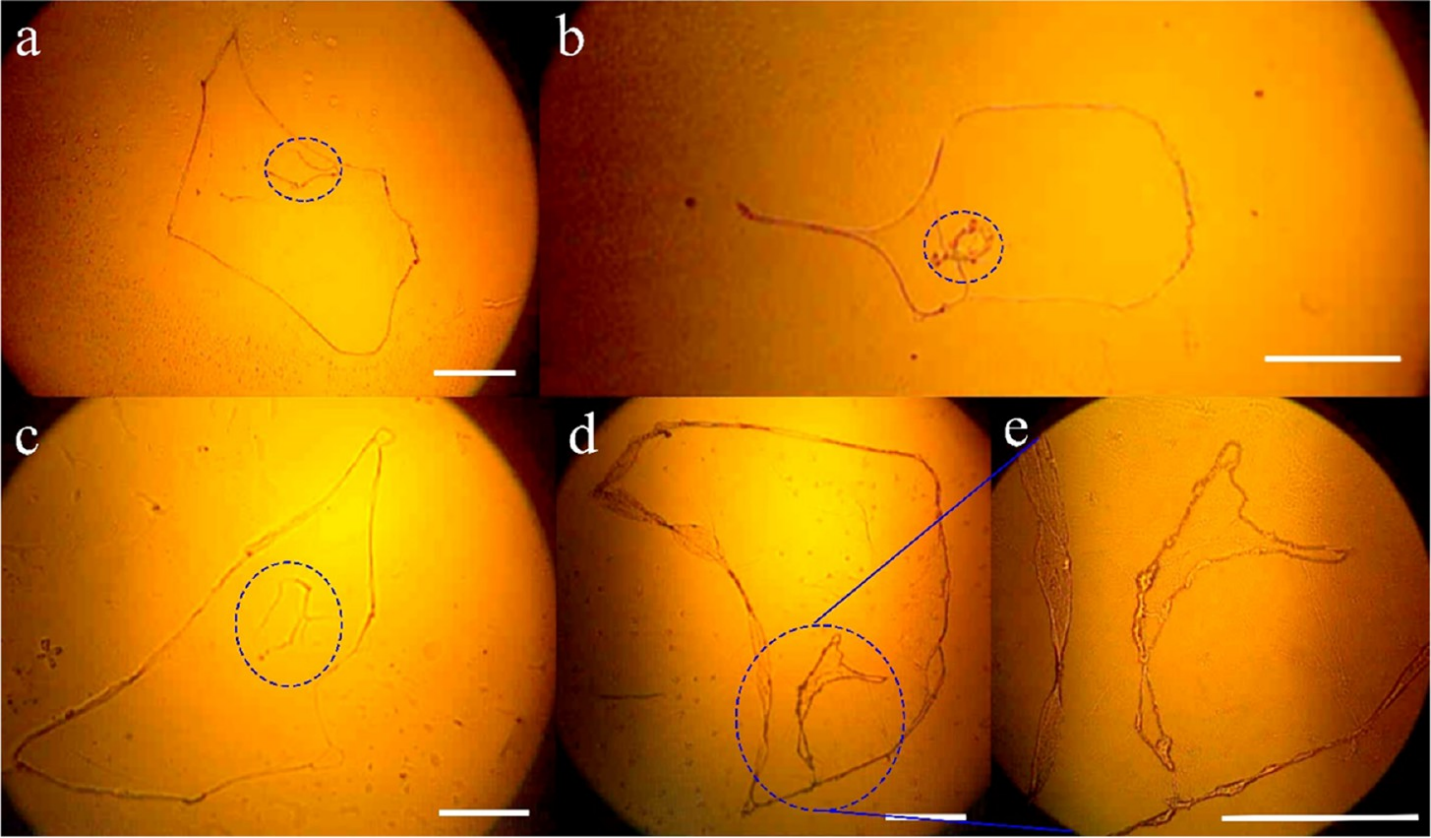
DNA was extracted from pregnant women carrying male fetuses, including (a) Subject 1 and (b) Subject 2 at 20 weeks of gestation, (c) Subject 3 at 13 weeks of gestation, and (d) Subject 3 at 40 weeks of gestation. (e) Amplified image from (d) corresponding to secondary strand. The precipitation condition used was 0.4–8–0.1. Image obtained through optical microscopy using a 4× objective.

Figure 15a,b shows two pregnant women in the 20th week of pregnancy, and Figure 15c,d shows the same pregnant woman in the 13th and 20th weeks of pregnancy. In the case of processed samples from pregnant women (Figure 15), structures have been systematically obtained that self-assemble separately when the DNA_ext_ precipitates on the slide. We assume that the larger chain or structure is associated with the DNA of the pregnant woman, while the smaller structure (blue circle) corresponds to the cfDNA of the pregnant product. Diverse researchers have reported the cfDNA of mother and fetus found in the bloodstream, and fetal cfDNA is shorter than maternal cfDNA.^71,72^

In both situations, the DNA hyperstructure is formed by a major strand and a minor strand. It is systematically observed that in pregnancies where the product is a boy, the secondary strand is confined within the region formed by the main strand. On the contrary, if the product of pregnancy is a girl, the secondary strand is attached to the main strand on the outside of the latter.

## Conclusions

The methodology proposed for extracting DNA from peripheral blood allows for the generation of extracts with high DNA concentrations (20–30 μg/μL). The use of BFC allows for the screening of repulsive electrostatic interactions between DNA strands, thus facilitating their self-assembly in precipitation with acid alcohol on a slide. The formation of structures upon precipitation of DNA_ext_ is marked by intense and localized DAPI emission, thereby ensuring nucleic acid participation in the precipitates. The millimeter-scale DNA structures, known as DNA hyperstructures, can be compressed or elongated through the regulation of the acid alcohol content during precipitation. The correlation between DNA concentration and the necessary AA_ga_ volume for precipitation is a linear, negative slope. As the DNA molecule is identical across all living organisms, this methodology permits the fabrication of DNA hyperstructures from any source of DNA and not exclusively from humans. We postulate that a comprehensive database could enable the detection of distinctive morphological patterns in DNA hyperstructures associated with individual diseases or pregnancies. Such patterns would enable the early identification of both diseases and pregnancies via a peripheral blood sample. For all diagnoses of pregnancy and disease, it is important to perform systematic studies that consider different stages of the clinical condition, which will lead us to understand the behavior of DNA hyperstructure in order to establish diagnoses at early stages and in a reliable manner. There is strong interest in understanding how methylation affects the geometric and mechanical properties associated with DNA folding and condensation. We believe that our methodology can contribute in this direction.

## Supporting Information Available

Protocol extraction and precipitation of: DNA healthy subjects (Table S1); DNA breast and uterine cervix cancer (Table S2); DNA pregnant woman (female fetuses) (Table S3); DNA pregnant woman (male fetuses) (Table S4); amplificated region with random coil structure of Figure 2 in bright-field mode and DAPI fluorescence emission (Figure S1); images of DAPI-stained DNA_ext_ (sample 1A-rep), using confocal microscopy in bright-field mode, DAPI fluorescence, and merged images (Figure S2); images of DNA_ext_ (sample 3A) DAPI-stained, using confocal microscopy in bright-field mode, DAPI fluorescence, and merged images; mosaic image was formed with 48 individual photographs obtained with a Plan-Apochromatic 40×/0.95 dry objective (Figure S3); optimal DNA hyperstructure for 28, 15, and 7 μg/μL DNA_ext_; for each case, the AA_ag_ volume optimal is 21, 23, and 24.5 μL; images were acquired using an optical microscope with a 4× objective (Figure S4); pBR322 DNA hyperstructure obtained by precipitation on the slide (1–200–5); DNA solution was prepared on BFC at 0.5 μg/μL; an optical microscope with a 4× objective captured the image (Figure S5); bright-field images merged with the fluorescent image associated with Figure 8; for precipitation DNA_ext_ volume maintained constant (1 μL) and AA_ga_ volumes were 20, 15, 10, and 7.5 μL (Figure S6); DNA hyperstructure using DAPI staining; the sample corresponds to a healthy 47-year-old woman; the bright-field image was captured in a conventional optical microscope with a 4× objective; the same sample was recorded by capturing DAPI fluorescence on a confocal microscope; DNA_ext_ was precipitated on the slide with parameters relation of 1–20–5 (Figure S7); and DNA hyperstructure of various subjects without chronic degenerative diseases; the scale corresponds to 500 μm (Figure S8) (PDF)
