# Supplementary material for: DNA Hyperstructure
Source: ACS Omega. 2024 Feb 12;9(8):9013–26. doi: 10.1021/acsomega.3c07379 (PMC10905968; doi:10.1021/acsomega.3c07379)
Supplement: Supplementary file 1 — ao3c07379_si_001.pdf [file ao3c07379_si_001.pdf]

Supporting Information

DNA Hyperstructure

[&*]Gloria Elena León-Paz-de-Rodríguez, [#*]Ericka Rodríguez-León, [#*]Ramón Iñiguez-Palomares

[&]Independent researcher, Hermosillo, Sonora, México. C.P. 83250

[#]Physics Department, Universidad de Sonora, Hermosillo, Sonora, México. C.P. 83250

corresponding author: gloriaelena.leon@gmail.com

corresponding author: ericka.rodriguez@unison.mx

corresponding author: ramon.iniguez@unison.mx

Healthy subjects				
R (μL)	BFC (μL)	DNA_{ext} (μL)	AA_{ga} (μL)	A_{ga} (%v)
40	4	1	10	5

Table S1. Protocol of extraction and precipitation of DNA hyperstructure

Breast Cancer					
R (μL)	BFC (μL)	DNA_{ext} (μL)	AA_{ga} (μL)	A_{ga} (%v)	condition
44	0	0.4	20	0.1	Treatment
Uterine Cervix Cancer					
44	0	0.4	20	0.1	Without Treatment

Table S2. Protocol of extraction and precipitation of DNA hyperstructure

Pregnancy female fetuses					
R (μL)	BFC (μL)	DNA_{ext} (μL)	AA_{ga} (μL)	A_{ga} (%v)	weeks
40	2	0.3	10	0.1	5
44	0	0.4	10	0.1	13
40	0	0.4	5	0.1	17
44	0	0.4	5	0.1	19

Table S3. Protocol of extraction and precipitation of DNA hyperstructure

Pregnancy male fetuses

R (μL)	BFC (μL)	DNA_{ext} (μL)	AA_{ga} (μL)	A_{ga} (%v)	weeks
44	0	0.6	10	0.1	17
44	0	0.4	8	0.1	20
40	2.5	1	10	5	40

Table S4. Protocol of extraction and precipitation of DNA hyperstructure

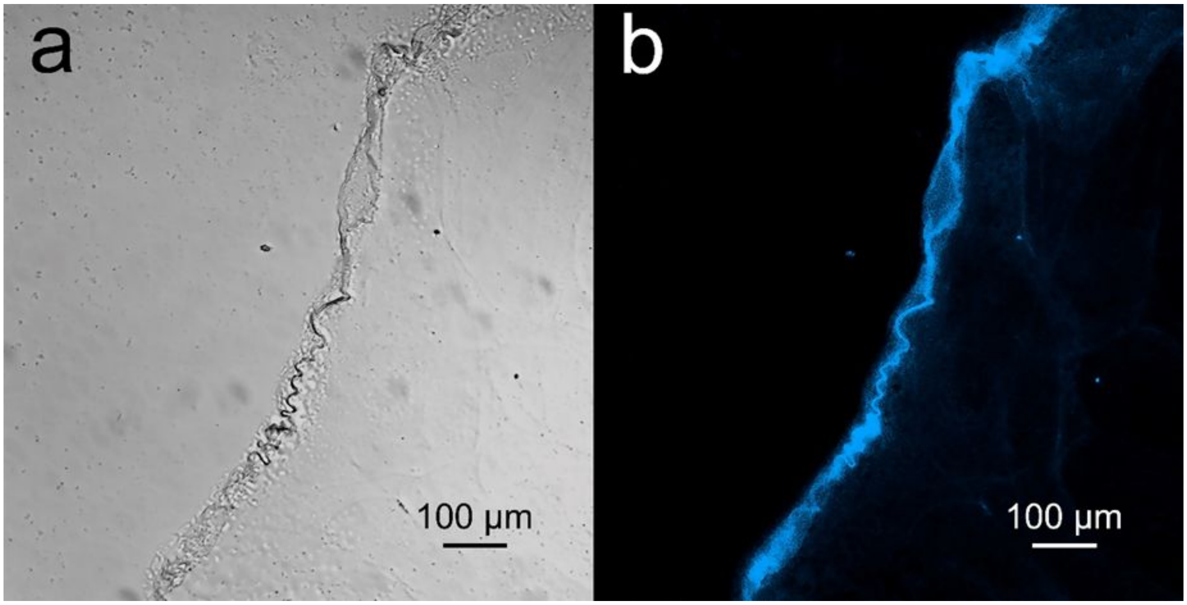

Figure S1. a) Amplificated region with random coil structure of Figure 2 in bright field mode, and b) DAPI fluorescence emission.

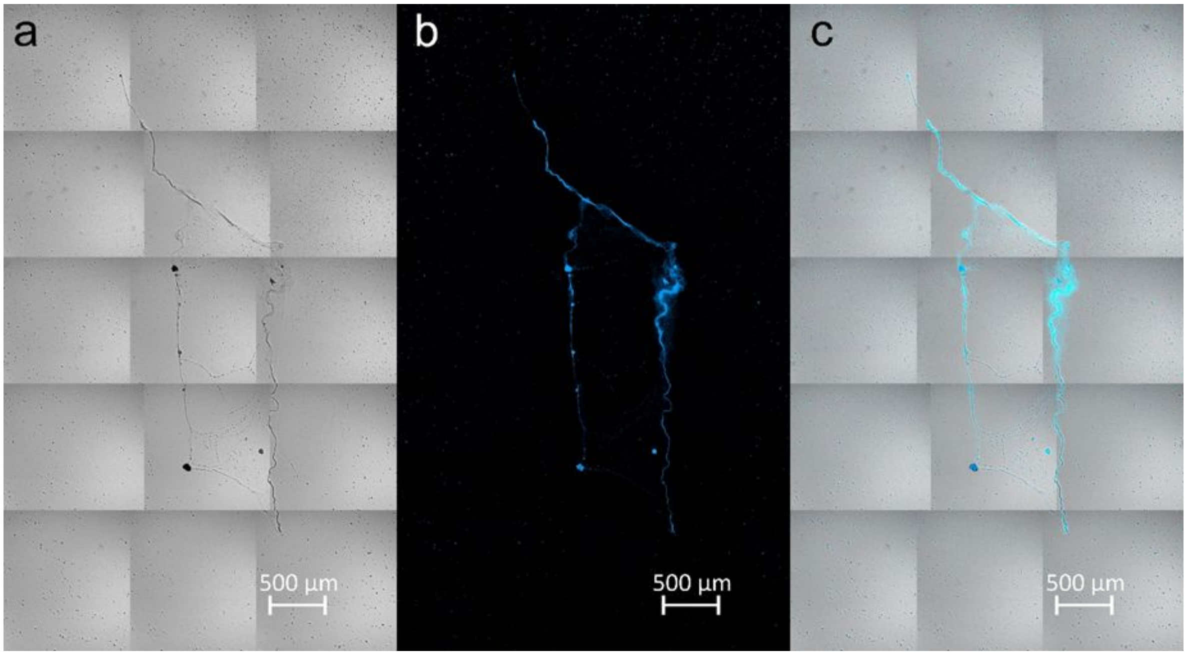

Figure S2. Images of DAPI stained DNA-extract (sample 1A-rep), using confocal microscopy in bright field mode (a), DAPI fluorescence (b), and merged images (c).

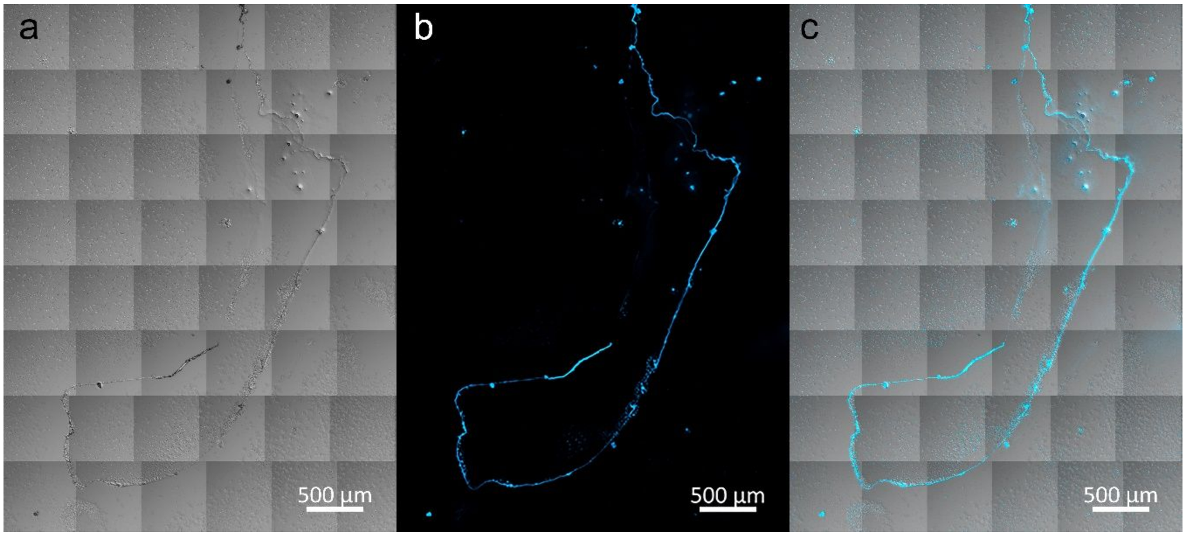

Figure S3. Images of DNA-extract (sample 3A) DAPI stained, using confocal microscopy in bright field mode (a), DAPI fluorescence (b), and merged images (c). Mosaic image was formed with 48 individual photographs obtained with a Plan-Apochromatic × 40/0.95 dry objective.

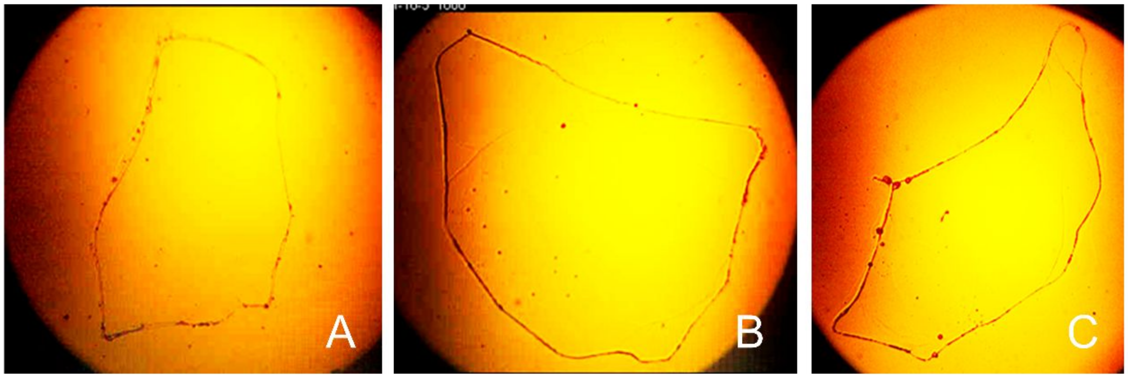

Figure S4. Optimal DNA hyperstructure for (A) 28, (B) 15, and (C) 7 μg/μL of DNA_{ext}. For each case, the AA_{ag} volume optimal is 21, 23, and 24.5 μL, respectively. Images were acquired using an optical microscope with a 4X objective.

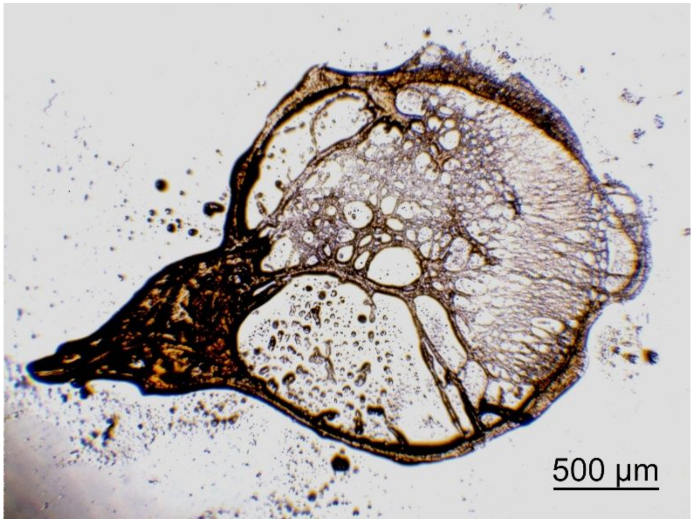

Figure S5. pBR322 DNA hyperstructure obtained by precipitation on the slide (1-200-5). The DNA solution was prepared on BFC at 0.5 µg/µL. An optical microscope with a 4X objective has captured the image.

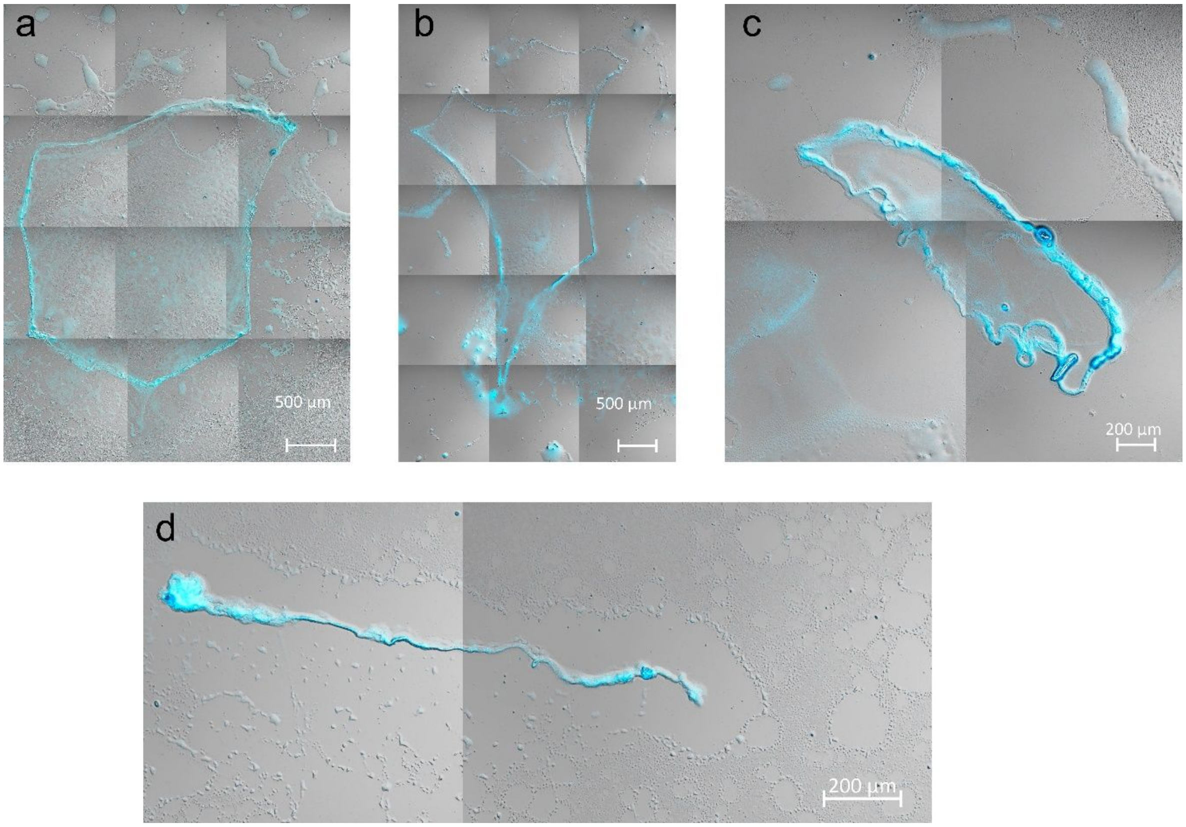

Figure S6. Brightfield images merged with the fluorescent image associated with Figure 8. For precipitation DNA_{ext} volume maintained constant (1 µL) and AA_{ga} volumes were 20, 15, 10, and 7.5 µL in a), b), c), and d), respectively.

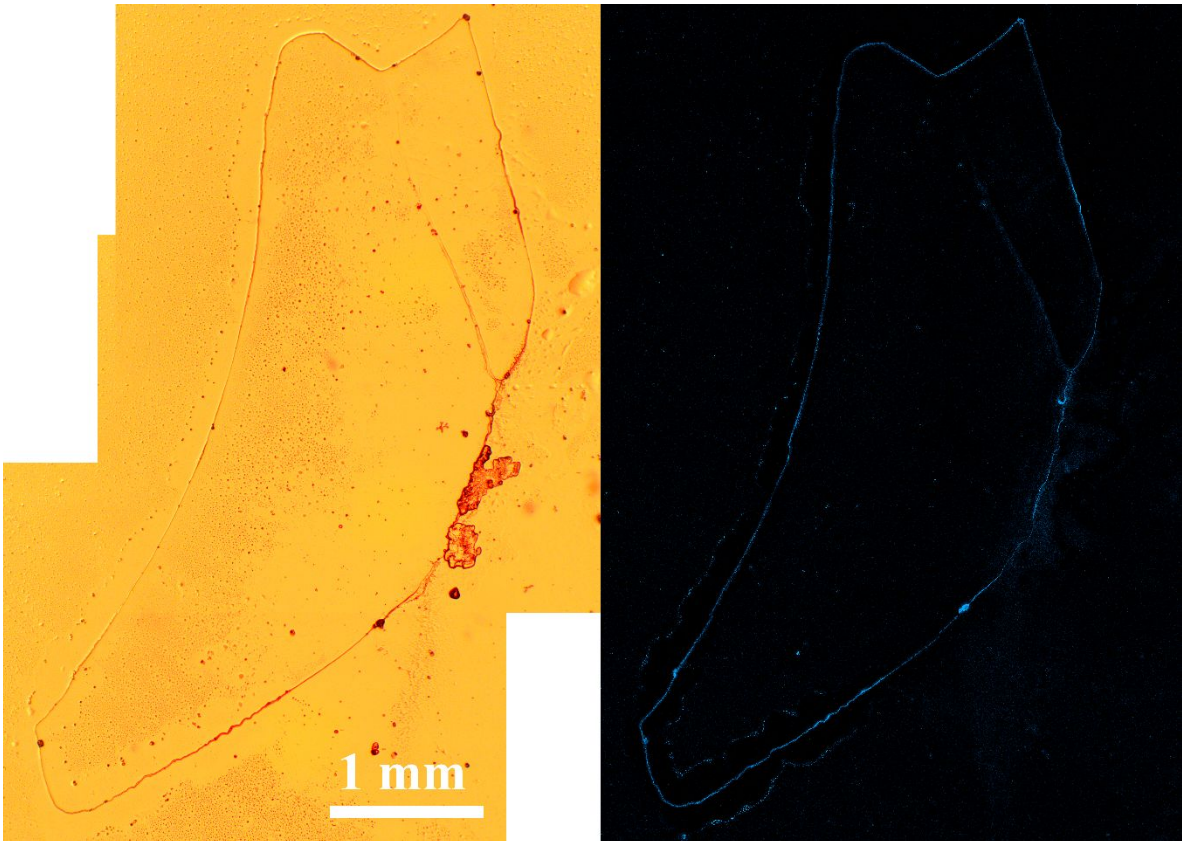

Figure S7. DNA hyperstructure using DAPI staining. The sample corresponds to a healthy 47-year-old woman. The bright field image was captured in a conventional optical microscope with a 4X objective (a). The same sample was recorded by capturing DAPI fluorescence on a confocal microscope (b). DNA_{ext} was precipitated on the slide with parameters relation of 1-20-5.

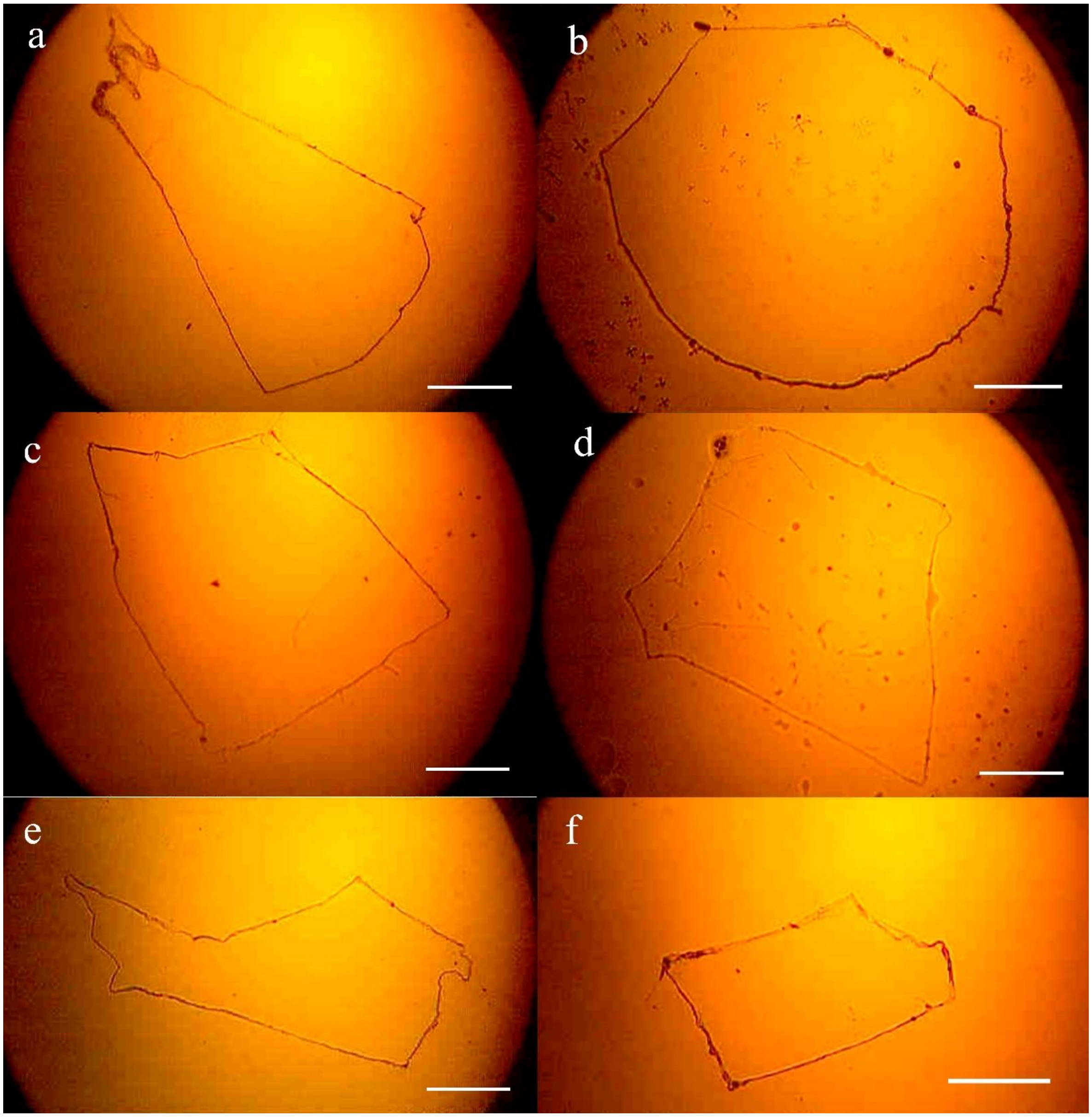

Figure S8. DNA hyperstructure of various subjects (a)-(f) without chronic-degenerative diseases. The scale corresponds to 500 µm.
